# Male Youth Tobacco Usage Pattern in Banned Smoking Area in Comparison With Non-banned Smoking Area: A Cross-Sectional Study

**DOI:** 10.7759/cureus.53503

**Published:** 2024-02-03

**Authors:** Abduallah Zaid M Alzaidy

**Affiliations:** 1 Family and Community Medicine, King Abdulaziz Medical City, Jeddah, SAU

**Keywords:** preventive measures, attitude, prevalence, saudi arabia, smoking ban area

## Abstract

Objectives: This study aimed to assess the impact of smoking bans in schools on smoking prevalence and behavior among Saudi male youth aged 13-15 years.

Methods: A cross-sectional comparative study was conducted involving students from two intermediate schools in Jeddah - one with a smoking ban and the other without. Data collection utilized the Global Youth Tobacco Survey questionnaire, and statistical analysis was performed using SPSS version 21.0.

Results: The study had a 93.9% response rate, with 659 students participating. Notably, a lower percentage of ever-smoking was observed in the banned area compared to the non-banned area (39.6% vs. 50.9%; p=0.002). Current smoking rates were also lower in the banned area (14.2% vs. 23.8%; p=0.014). Family and peer influences on smoking were reduced in the banned area, and more students discussed the harmful effects of smoking with family (72.8% vs. 59.8%; p=0.003). Students in the non-banned area found it easier to access cigarettes. A significantly higher percentage of students in the banned area were resolute in not smoking if offered a cigarette by their best friend (65.0% vs. 59.2%; p=0.006). Students in the non-banned area reported higher exposure to cigarette smoke at home and in other places compared to those in the banned area (15.8% vs. 10.8%; p=0.008), respectively. A higher percentage of smokers in the banned area expressed a desire to quit smoking, though the difference was not statistically significant. More anti-smoking media messages were reported in the banned area (35.6% vs. 33.6%; p=0.004). Fewer respondents in the banned area had items with cigarette brand logos (13.6% vs. 19.9%; p=0.03).

Conclusion: The findings underscore the effectiveness of smoking bans in schools in reducing smoking prevalence among students. This suggests a broader societal shift in attitudes toward smoking, highlighting the need for comprehensive bans as part of public health strategies. However, there remains a need for targeted interventions to address the complexities of smoking behavior in both banned and non-banned areas.

## Introduction

Smoking is one of the most common preventable causes of morbidity and premature death. About 1.3 billion tobacco users are there worldwide and this is expected to increase to 1.5 billion by the end of 2025 [1,2]. There are seven million deaths due to smoking-related causes every year, and every four seconds, there is another death [3-5]. Smoking prevalence is higher in countries with high income [1]. The most common causes of death related to smoking are cancer, chronic obstructive pulmonary disease, coronary artery disease, and stroke, and we can reduce their prevalence by decreasing the spread of smoking behavior [6].

Youths who are less than 15 years old are more susceptible to smoking [7]. Many studies reported that most smokers start smoking at the age of 15 years or earlier, and they attribute this to several reasons, including parental, sibling, and friends smoking, easy access to cigarettes, exposure to smoking behavior in social media, and socioeconomic status [7-10]. Smoking at a young age is associated with an increased risk of asthma and impaired lung function, risk of hypertension, cardiovascular diseases, impaired brain development, premature death, and addiction and reduces the likelihood of quitting smoking in the future [11-13]. On the other hand, the life expectancy of smokers who give up before their mid-30s will be similar to that of never smoking. Smoking cessation shows a decrease in the risk of smoking-related diseases [6,14]

This global context of smoking and its impacts sets the stage for examining the situation in specific regions, such as Saudi Arabia. In recent years, Saudi Arabia has experienced a noticeable rise in smoking prevalence. Over the last few years, Saudi Arabia has witnessed a significant increase in the prevalence of smoking. World Health Organization (WHO) reported that the prevalence of smoking in Saudi Arabia was 14% in 2013 [15]. Many studies assessed the prevalence of smoking in the period from 2009 to 2015 and found that the prevalence of smoking in Saudi Arabia ranged from 12.2% to 16.9% [16]. The spreading of smoking behavior in Saudi Arabia is a serious health problem due to the burden of the associated adverse events. Therefore, many studies measured the prevalence of smoking among youths and associated risk factors to improve our understanding of the issue's burden and to find approaches to prevent its spread in future generations.

In 2002, a survey based on the Global Youth Tobacco Survey (GYTS) was conducted in Riyadh to assess the prevalence of smoking among male school students aged from 13 to 15 years. The main results of this survey were that about 34.5% of the participants have ever smoked in their lives, 22.2% are current smokers, and 6.7% think of trying smoking through the next year [17]. When comparing these results with the same survey conducted in 2007, we notice an increased number of male students who have tried smoking 39.5%, the number of male students currently smoking to 13%, and the number who are thinking of trying smoking through the next year to 20.7% [18].

To control the spread of smoking, the Saudi Arabian government has started several programs in schools to raise awareness of the dangers and harms of smoking. These programs included increased lectures about the hazards of smoking, anti-smoking media messages, awareness lectures for parents, and posters [19]. Following these measures, a slight reduction in smoking prevalence was observed through a survey conducted in 2010; about 34.6% of the participants had ever smoked in their life, 21.2% were current smokers, and there was an increase in the prevalence of the number of the participants who think of trying smoking through the next year to 21.2% [20]. To combat the rising smoking rates in Saudi Arabia, prevention programs still require improvement. In our study, we aimed to describe smoking patterns among Saudi male youth aged 13-15 in two intermediate schools in Jeddah in 2011 through the GYTS survey.

## Materials and methods

Study design and population

This cross-sectional comparative study was conducted to explore the impact of smoking bans on the smoking behaviors and attitudes of intermediate school students in Saudi Arabia. The study focused on students from two schools: the Abu Obeida Intermediate School in King Faisal Housing City (KFHC), where a smoking ban has been in effect since 2002, and the Raja bin Hyouah Intermediate School in Bahra district, where no such ban exists. This setting provided a unique opportunity to compare the influence of smoking policies on a young population.

The study included Saudi students aged between 13 and 15 years from these selected schools. We deliberately chose this age group as it represents a critical period in adolescence when children are more likely to experiment with smoking. Inclusion criteria were strictly adhered to, and we excluded non-Saudi students. This exclusion was based on the need to maintain a homogeneous study population and to respect the personal choices of the students.

Before the commencement of the study, ethical approval was diligently obtained from the Joint Program of Family & Community Medicine (IRB: JP21R/022/01). This step was crucial to ensure that the study adhered to ethical standards and protected the rights and well-being of the participants.

To ensure the reliability and validity of the data collected, participants were assured that their responses would remain confidential. Additionally, written informed consent was obtained from all participants and their guardians. This consent process was conducted in accordance with ethical guidelines and served to inform participants and their families about the nature, benefits, and potential risks of the study.

Data collection and questionnaire

The study participants were selected using a random sampling method. This self-reporting questionnaire was distributed among eligible participants and their responses were collected. Data collection for this study was meticulously carried out using the Arabic version of the GYTS questionnaire, a comprehensive and widely recognized tool in tobacco-related research [21]. This version comprises 56 core questions, meticulously crafted to cover a broad spectrum of topics related to tobacco use. The questionnaire's content spans from personal smoking habits to perceptions and knowledge about smoking, making it a robust instrument for gathering detailed data. Notably, the GYTS questionnaire is published under the Creative Commons License (CC-BY). This licensing allows for unrestricted use, distribution, and adaptation of the questionnaire, ensuring its wide applicability and facilitating its adoption in varied contexts. Importantly, the questionnaire's reliability and validity were rigorously tested. A subset of participants, representing 10% of the total sample, was selected to assess the questionnaire's reliability. This assessment yielded a correlation coefficient of 0.89, a strong indication of its reliability and the consistency of responses. Additionally, the internal consistency of the questionnaire was evaluated, resulting in a Cronbach's alpha value of 0.78. These metrics collectively underscore the instrument's high level of dependability and accuracy in measuring the constructs of interest.

The questionnaire was designed to delve deeply into several key areas, providing a comprehensive understanding of the smoking landscape among the adolescent population. It explored not only the prevalence of cigarette smoking and other forms of tobacco use but also probed into the student's knowledge and attitudes toward smoking. This included assessing their awareness of the health risks associated with smoking and their perceptions of the social acceptability of the habit. The influence of media and advertising on smoking behaviors was another critical area of inquiry, considering the pervasive nature of these mediums in shaping youth attitudes and behaviors. Moreover, the questionnaire investigated the accessibility of cigarettes to the students and the extent to which tobacco-related topics were covered in their school curriculum. Exposure to secondhand smoke and the efforts and intentions to quit smoking were also integral components of the survey, providing insights into the broader environmental and personal factors influencing tobacco use. To ensure the effective administration of the questionnaire, it was distributed to students in their classrooms, where they could record their answers directly. This approach ensured a controlled environment and minimized external influences on the respondents. 

Data analysis

The Statistical Package for Social Sciences (SPSS; version 21.0, IBM Corp, Armonk, NY) was used to analyze the data. The quantitative and categorical variables were described using descriptive statistics, specifically mean, standard deviation, and percentages. The chi-squared test was used to compare the distribution of categorical variables. Significance was defined as a p-value less than 0.05.

## Results

Demographic characteristics of included students

**Figure 1:**
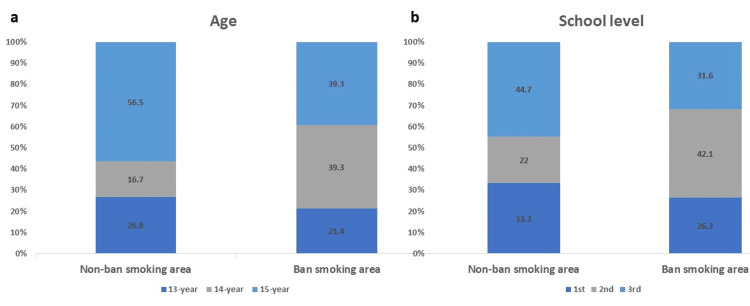
Study population distribution according to a) age, b) school level

We collected responses from 659 students: 323 from Abu Obeida Intermediate School (banned smoking area) and 336 from Raja bin Hyouah Intermediate School (non-banned smoking area). The overall response rate was 93.9% (94.4% in the banned area and 93.3% in the non-banned area). The majority of respondents were 15 years old (56.5% in the banned area and 39.3% in the non-banned area), as shown in Figure 1a. Regarding school-level distribution, approximately 31.6% in the banned area and 44.7% in the non-banned area were in the third intermediate level, as shown in Figure 1b.

Prevalence of smoking

The prevalence of smoking was notably lower in the banned area, with 39.6% of students having ever smoked cigarettes compared to 50.9% in the non-banned area (p=0.002), and current cigarette smoking was also lower (14.2% vs. 23.8%, p=0.014). However, there was no significant difference in the prevalence of ever-smoking Shisha (26.0% vs. 32.1%, p=0.083). Smoking frequency also varied, with only 0.3% of students in the banned area smoking during all days of the past month, compared to 5.7% in the non-banned area (p=0.006). The age of initiating smoking did not show significant differences across different age brackets (p=0.584). In terms of family influence, fewer students in the banned area reported having parents who smoke (70.9% vs. 75.9%, p=0.007). Additionally, fewer students in the banned area had big brothers (25.7% vs. 36.0%, p=0.001) who smoke. This trend extended to peer influence, with fewer students in the banned area reporting that some of their closest friends smoke (27.2% vs. 37.5%, p<0.001). Moreover, a higher percentage of students in the banned area were resolute in not smoking if offered a cigarette by their best friend (65.0% vs. 59.2%, p=0.006). However, parental awareness of their child's smoking habits did not differ significantly between areas (14.3% in the banned area vs. 25.0% in the non-banned area, p=0.887). Interestingly, more students in the banned area reported having family discussions about the harmful effects of smoking (72.8% vs. 59.8%, p=0.003), as shown in Table 1.

**Table 1:** Prevalence of smoking among students and their relatives or friends

Variables		Total (n=659); n (%)	Banned smoking area (n=323); n (%)	Non-banned smoking area (n=336); n (%)	p-Value
Prevalence of smoking among students	Ever smoked cigarettes	299 (45.4)	128 (39.6)	171 (50.9)	0.002
	Current cigarette smokers	126 (19.1)	46 (14.2)	80 (23.8)	0.014
	Ever smoked Shisha	192 (29.1)	84 (26.0)	108 (32.1)	0.083
Smoking during all days of the past month		20 (3.0)	1 (0.30)	19 (5.7)	0.006
Age when first start smoking	Never smoked shisha*	467 (70.9)	239 (74)	228 (67.9)	0.584
	≤7 years	31 (4.7)	15 (4.6)	16 (4.8)	
	8-9 years	28 (4.3)	12 (3.7)	16 (4.8)	
	10-11 years	39 (5.9)	18 (5.6)	21 (6.2)	
	12-13 years	46 (7.0)	18 (5.6)	28 (8.3)	
	14-15 years	48 (7.2)	21 (6.5)	27 (8.0)	
During the past month. How many cigarettes did you usually smoke/day?	Did not smoke during last month*	533 (80.9)	277 (85.8)	256 (76.2)	0.162
	<1	31 (4.7)	13 (4.0)	18 (5.4)	
	1	32 (4.9)	16 (5.0)	16 (4.8)	
	2-5	36 (5.5)	11 (4.5)	25 (7.4)	
	6-10	16 (2.4)	5 (1.4)	11 (3.3)	
	11-20	4 (0.6)	1 (0.3)	3 (0.9)	
	>20	7 (1.1)	0 (0.0)	7 (2.0)	
Prevalence of smoking in families and friends					
Do your parents’ smoke?	None	484 (73.4)	229 (70.9)	255 (75.9)	0.007
	Both	9 (1.4)	1 (0.3)	8 (2.4)	
	Father only	149 (22.6)	83 (25.7)	66 (19.6)	
	Mother only	3 (0.5)	0 (0.0)	3 (0.9)	
	I don't know	14 (2.1)	10 (3.1)	4 (1.2)	
Does your uncle smoke? (Yes)	Yes	347 (52.7)	190 (58.9)	157 (46.7)	0.006
	No	259 (39.3)	108 (33.4)	151 (44.9)	
	I don't know	53 (8.0)	25 (7.7)	28 (8.4)	
Does your big brother smoke?	Yes	204 (31.0)	83 (25.7)	121 (36.0)	0.001
	No	422 (64.0)	229 (70.9)	193 (57.4)	
	I don't know	33 (5.0)	11 (3.4)	22 (6.6)	
Do any of your closest friends smoke cigarettes?	None of them	332 (50.4)	193 (59.8)	139 (41.4)	<0.001
	Some of them	214 (32.5)	88 (27.2)	126 (37.5)	
	Most of them	76 (11.5)	31 (9.6)	45 (13.4)	
	All of them	37 (5.6)	11 (3.4)	26 (7.7)	
If one of your best friends offered you a cigarette, would you smoke it?	Definitely not	409 (62.1)	210 (65.0)	199 (59.2)	0.006
	Probably not	91 (13.8)	53 (16.4)	38 (11.3)	
	Probably yes	93 (14.1)	37 (11.5)	56 (16.7)	
	Definitely yes	66 (10.0)	23 (7.1)	43 (12.8)	
Do your parents know that you are smoker? (Yes)		39 (5.9)	12 (14.3)	27 (25.0)	0.887
Has anyone in your family discussed the harmful effects of smoking with you? (Yes)		436 (66.2)	235 (72.8)	201 (59.8)	0.003

Accessibility and attitudes

Students in the non-banned area found it easier to access cigarettes compared to their counterparts in the banned area. This was evident both in purchasing cigarettes (11.6% in the non-banned area vs. 6.5% in the banned area) and in instances of stealing them (6.5% vs. 1.5%, respectively), with the differences being statistically significant (p=0.003), as shown in Table 2.

**Table 2:** Accessibility to tobacco

Variables		Total (n=659); n (%)	Banned smoking area (n=323); n (%)	Non-banned smoking area (n=336); n (%)	p-Value
During the past month, how did you get your cigarettes?	Did not smoke during the last month*	533 (80.9)	277 (85.8)	256 (76.2)	0.003
	Store, shop, or street vendor	60 (9.1)	21 (6.5)	39 (11.6)	
	Vending machine	3 (0.5)	3 (0.9)	0 (0.0)	
	Someone else bought it for me	3 (0.5)	2 (0.6)	1 (0.3)	
	Borrowed them from someone else	10 (1.5)	8 (2.6)	2 (0.6)	
	Stole them	27 (4.1)	5 (1.5)	22 (6.5)	
	From older person	4 (0.6)	2 (0.6)	2 (0.6)	
	Other ways	19 (2.9)	5 (1.5)	14 (4.2)	
During the past month, did anyone refuse to sell you cigarettes?	I did not try to buy cigarettes*	519 (78.8)	263 (81.4)	256 (76.2)	0.214
	Yes, because of my age	57 (8.7)	28 (8.7)	29 (8.6)	
	No, my age did not keep me	83 (12.6)	32 (9.9)	51 (15.2)	

In areas with a smoking ban, a significantly higher percentage of individuals (68.7% vs. 58.3%, p=0.02) believed they would not smoke in the next 12 months, and a similar trend was observed for smoking expectations over the next five years (68.4% vs. 59.8%, p=0.016). When considering social perceptions, respondents in the banned area were more likely to think that boys who smoke have more friends (40.6% vs. 31.5%, p<0.001) and that smoking makes boys look more attractive (23.5% vs. 12.5%, p<0.001). However, the belief that smoking makes girls look more attractive was less prevalent in the banned area (12.7% vs. 20.8%, p=0.007). In terms of health perceptions, a larger proportion in the banned area recognized the harmful effects of smoking (74% vs. 71.4%, p=0.029). Interestingly, there were no significant differences in the perception of the difficulty of quitting smoking between the two areas (p=0.170). Regarding the social utility of smoking, fewer respondents in the banned area believed that smoking makes one more comfortable in social settings (19.5% vs. 25.0%, p=0.101). There were also notable differences in the perception of smokers, with more respondents in the non-banned area viewing smoking men as lacking confidence (21.7% vs. 16.1%, p=0.007) and stupid (20.5% vs. 26.3%, p=0.049). Finally, the belief in the safety of short-term smoking was significantly lower in the banned area (50.7% vs. 41.1%, p=0.005), as shown in Table 3.

**Table 3:** Attitudes of students toward tobacco smoking

Variables		Total (n=659); n (%)	Banned smoking area (n=323); n (%)	Non-banned smoking area (n=336); n (%)	p-Value
During the next 12 months do you think you will smoke a cigarette?	Definitely No	418 (63.4)	222 (68.7)	196 (58.3)	0.02
	Probably No	130 (19.7)	59 (18.3)	71 (21.1)	
	Probably Yes	61 (9.3)	26 (8.0)	35 (10.4)	
	Definitely Yes	50 (7.6)	16 (5.0)	34 (10.2)	
Do you think you will be smoking cigarettes 5 years from now?	Definitely No	422 (64.0)	221 (68.4)	201 (59.8)	0.016
	Probably No	133 (20.2)	60 (18.6)	73 (21.7)	
	Probably Yes	69 (10.5)	33 (10.2)	36 (10.7)	
	Definitely Yes	35 (5.3)	9 (2.8)	26 (7.8)	
Do you think boys who smoke cigarettes have more or less friends?	More friends	237 (36.0)	131 (40.6)	106 (31.5)	<0.001
	Fewer friends	156 (23.7)	90 (27.9)	66 (19.6)	
	No difference from non-smokers	41 (6.2)	13 (4.0)	28 (8.3)	
	I don't know	225 (34.1)	89 (27.5)	136 (40.6)	
Do you think smoking cigarettes makes boys look more or less attractive?	More attractive	118 (17.9)	76 (23.5)	42 (12.5)	<0.001
	Less attractive	182 (27.6)	107 (33.1)	75 (22.3)	
	No difference from non-smokers	57 (8.7)	10 (3.1)	47 (14.0)	
	I don't know	302 (45.8)	130 (40.3)	172 (51.2)	
Do you think smoking cigarettes makes girls look more or less attractive?	More attractive	111 (16.8)	41 (12.7)	70 (20.8)	0.007
	Less attractive	192 (29.1)	109 (33.8)	83 (24.7)	
	No difference from non-smokers	30 (4.6)	12 (3.7)	18 (5.4)	
	I don't know	326 (49.5)	161 (49.8)	165 (49.1)	
Do you think cigarette smoking is harmful to your health?	Definitely No	64 (9.7)	28 (8.7)	36 (10.7)	0.029
	Probably No	33 (5.0)	9 (2.8)	24 (7.1)	
	Probably Yes	83 (12.6)	47 (14.5)	36 (10.7)	
	Definitely Yes	479 (72.7)	239 (74)	240 (71.4)	
Once someone has started smoking, do you think it would be difficult to quit?	Definitely No	292 (44.3)	152 (47.1)	140 (41.7)	0.170
	Probably No	201 (30.5)	101 (31.3)	100 (29.7)	
	Probably Yes	98 (14.9)	44 (13.6)	54 (16.1)	
	Definitely Yes	68 (10.3)	26 (13.0)	42 (12.5)	
Does smoking cigarettes help people feel more or less comfortable at celebrations, parties, or in other social gatherings?	More comfortable	147 (22.3)	63 (19.5)	84 (25.0)	0.101
	Less comfortable	400 (60.7)	211 (65.3)	189 (56.3)	
	No difference from non-smokers	11 (1.7)	5 (1.5)	6 (1.8)	
	I don’t know	101 (15.3)	44 (13.7)	57 (16.9)	
Do you think that smoking cigarettes makes you gain or lose weight?	Gain weight	58 (8.8)	33 (10.2)	25 (7.4)	0.481
	Lose weight	209 (31.7)	102 (31.6)	107 (31.8)	
	No difference	72 (10.9)	31 (9.6)	41 (12.2)	
	I don’t know	320 (48.6)	157 (48.6)	163 (48.6)	
When you see a man smoking what do you think of him?	Lacks confidence	125 (19.0)	52 (16.1)	73 (21.7)	0.007
	Stupid	114 (17.3)	70 (21.7)	44 (13.1)	
	Loser	94 (14.3)	43 (13.3)	51 (15.2)	
	Successful	18 (2.7)	4 (1.2)	14 (4.2)	
	Intelligent	13 (2.0)	7 (2.2)	6 (1.7)	
	He is a "real man"	18 (2.7)	7 (2.2)	11 (3.3)	
	Disobey his religious teaching	277 (42.0)	140 (4.3)	137 (40.8)	
When you see a man smoking what do you think of him?	Lacks confidence	92 (14.0)	33 (10.2)	59 (17.6)	0.049
	Stupid	154 (23.4)	85 (26.3)	69 (20.5)	
	Loser	119 (18.1)	51 (15.8)	68 (20.2)	
	Successful	14 (2.1)	8 (2.5)	6 (1.8)	
	Intelligent	14 (2.1)	6 (1.9)	8 (2.4)	
	He is a "real man"	18 (2.7)	9 (2.8)	9 (2.7)	
	Disobey his religious teaching	248 (37.6)	131 (40.5)	117 (34.8)	
Do you think it is safe to smoke for only a year or two as long as you quit after that?	Definitely not	302 (45.8)	164 (50.7)	138 (41.1)	0.005
	Probably not	157 (23.8)	69 (21.4)	88 (26.2)	
	Probably yes	60 (9.1)	20 (6.2)	40 (11.9)	
	Definitely yes	27 (4.1)	9 (2.8)	18 (5.3)	
	I don't know	113 (17.2)	61 (18.9)	52 (15.5)	

Passive smoking

Students in the non-banned area reported higher exposure to cigarette smoke at home (15.8%) compared to those in the banned area (7.8%; p<0.001). Exposure to smoke from others' cigarettes in places other than home was also higher in the non-banned area (15.8% vs 10.8%, p=0.008). Moreover, there was a stronger agreement on banning smoking in public places among students in the banned area (69.3%) compared to the non-banned area (53.6%; p<0.001), as shown in Table 4.

**Table 4:** Passive smoking

Variables		Total (n=659); n (%)	Banned smoking area (n=323); n (%)	Non-banned smoking area (n=336); n (%)	p-Value
Do you think the smoke from other people’s cigarettes is harmful to you?	Definitely No	75 (11.4)	25 (7.8)	50 (14.9)	0.015
	Probably No	47 (7.1)	19 (5.9)	28 (8.3)	
	Probably Yes	179 (27.2)	94 (29.1)	85 (25.3)	
	Definitely Yes	358 (54.3)	185 (57.2)	173 (51.5)	
During the past 7 days, on how many days have people smoked in your home, in your presence?	Zero	378 (57.4)	208 (64.4)	170 (50.6)	<0.001
	1 to 2	127 (19.3)	55 (17.0)	72 (21.4)	
	3 to 4	41 (6.2)	17 (5.3)	24 (7.1)	
	5 to 6	25 (3.8)	8 (2.5)	17 (5.1)	
	7	88 (13.4)	35 (10.8)	53 (15.8)	
During the past 7 days, on how many days have people smoked in your presence, in places other than in your home?	Zero	378 (57.4)	208 (64.4)	170 (50.6)	0.008
	1 to 2	127 (19.3)	55 (17.0)	72 (21.4)	
	3 to 4	41 (6.2)	17 (5.3)	24 (7.1)	
	5 to 6	25 (3.8)	8 (2.5)	17 (5.1)	
	7	88 (13.4)	35 (10.8)	53 (15.8)	
Are you in favor of banning smoking in public places (such as in restaurants, buses, streetcars, and trains, in schools, on playgrounds, in gyms and sports arenas, in discos)?		404 (61.3)	224 (69.3)	180 (53.6)	<0.001

Cessation among current smokers

In the banned area, a higher percentage of smokers expressed a desire to quit smoking (67.4%) compared to the non-banned area (53.8%), though this difference was not statistically significant (p=0.134). Similarly, the proportion of smokers who had attempted to quit in the past year was slightly higher in the banned area (60.9% vs. 56.2%), but this difference was also not statistically significant (p=0.613). The time since quitting smoking showed a varied distribution across both areas, with no significant differences in terms of 1-3 months, 4-11 months, one year, two years, or three years or longer (p=0.856). The main reasons for deciding to stop smoking were predominantly to improve health in both areas (60.7% in the banned area vs. 66.7% in the non-banned area, p=0.448), with other reasons being less common. Notably, a significantly higher proportion of smokers in the banned area believed they could stop smoking if they wanted to (76.1% vs. 56.2% in the non-banned area, p=0.026). In terms of receiving help or advice to quit smoking, there was a higher incidence of assistance from programs or professionals in the banned area (34.8% vs. 16.2%), although this difference was not statistically significant (p=0.165), as shown in Table 5.

**Table 5:** Attitudes toward quitting smoking among current smokers

Variables		Total (n=126); n (%)	Banned smoking area (n=46); n (%)	Non-banned smoking area (n=80); n (%)	p-Value
Do you want to stop smoking now?	Yes	74 (58.7)	31 (67.4)	43 (53.8)	0.134
	No	52 (41.3)	15 (32.6)	37 (36.2)	
During the past year, have you ever tried to stop smoking cigarettes?	Yes	73 (58.0)	28 (60.9)	45 (56.2)	0.613
	No	53 (42.1)	18 (39.1)	35 (43.8)	
How long ago did you stop smoking?	1-3 months	26 (20.6)	9 (32.1)	17 (37.8)	0.856
	4-11 months	7 (5.7)	2 (7.1)	5 (11.2)	
	One year	9 (7.1)	3 (10.7)	6 (13.3)	
	2 years	12 (9.5)	6 (21.4)	6 (13.3)	
	3 years or longer	19 (15.1)	8 (28.6)	11 (24.4)	
What was the main reason you decided to stop smoking?	To improve my health	47 (37.3)	17 (60.7)	30 (66.7)	0.448
	To save money	3 (2.4)	1 (3.5)	2 (4.4)	
	Because my family does not like it	9 (7.1)	5 (17.9)	4 (8.9)	
	Because my friends do not like it	2 (1.6)	0 (0.0)	2 (4.4)	
	Others	12 (9.5)	5 (17.9)	7 (15.6)	
Do you think you would be able to stop smoking if you wanted to?	Yes	80 (63.5)	35 (76.1)	45 (56.2)	0.026
	No	46 (36.5)	11 (23.9)	35 (43.8)	
Have you ever received help or advice to help you stop smoking?	Yes, from a program or professional	29 (23.0)	16 (34.8)	13 (16.2)	0.165
	Yes, from a friend	35 (27.8)	9 (19.6)	26 (32.5)	
	Yes, from a family member	22 (17.5)	8 (17.4)	14 (17.5)	
	Yes, from both programs and professionals	17 (13.5)	6 (13.0)	11 (13.8)	
	No	23 (18.3)	7 (15.2)	16 (20.0)	

Media and school anti-smoking activities

Regarding media activities, a higher percentage of respondents in the banned area reported seeing a lot of anti-smoking media messages in the past month (35.6% vs. 33.6%, p=0.004). Additionally, in the banned area, more people frequently encountered anti-smoking messages at sports events, community events, or social gatherings (52.2% vs. 42.9%, p=0.013). However, the frequency of seeing actors smoking on TV, in videos, or in movies was similar in both areas (p=0.528). Fewer respondents in the banned area had items with cigarette brand logos (13.6% vs. 19.9%, p=0.03), and there was no significant difference in seeing cigarette brand names on TV (p=0.65) or cigarette advertisements on billboards (p=0.233). Interestingly, there were significantly more advertisements or promotions for cigarettes seen in newspapers or magazines in the banned area (31.3% vs. 27.4%, p<0.001). In terms of school activities, fewer respondents in the banned area reported that a teacher or another person talked in class about the dangers of smoking (37.8% vs. 46.1%, p=0.041). There was also less discussion about the reasons why people their age smoke (18.0% vs. 30.7%, p=0.001) and about the effects of smoking (22.6% vs. 38.1%, p<0.001) in the banned area compared to the non-banned area, as shown in Table 6.

**Table 6:** Media and school anti-smoking activities

Variables			Total (n=659); n (%)	Banned smoking area (n=323); n (%)	Non-banned smoking area (n=336); n (%)	p-Value
	Media activities for anti-smoking					
During the past month, how many anti-smoking media messages have you seen or heard?		A lot	228 (34.6)	115 (35.6)	113 (33.6)	0.004
		A few	193 (29.3)	110 (34.1)	83 (24.7)	
		None	238 (36.1)	98 (30.3)	140 (41.7)	
When you go to sports events, community events, or social gatherings, how often do you see anti-smoking messages?		I never go to sports events, fairs, concerts, community events, or social gatherings	121 (18.4)	48 (14.9)	73 (21.7)	0.013
		A lot	313 (47.5)	169 (52.2)	144 (42.9)	
		Sometimes	109 (16.5)	58 (18.0)	51 (15.2)	
		Never	116 (17.6)	48 (14.9)	68 (20.2)	
When you watch TV, videos, or movies, how often do you see actors smoking?		I never watch TV, videos, or movies	68 (10.3)	31 (9.6)	37 (11.0)	0.528
		A lot	425 (64.5)	215 (66.6)	210 (62.5)	
		Sometimes	112 (17.0)	55 (17.0)	57 (17.0)	
		Never	54 (8.2)	22 (6.8)	32 (9.5)	
Do you have something (t-shirt, pen, backpack, etc.) with a cigarette brand logo on it?		Yes	111 (16.8)	44 (13.6)	67 (19.9)	0.03
		No	548 (83.2)	279 (86.4)	269 (80.1)	
During the one month, when you watched programs on TV how often did you see cigarette brand names?		I never watch TV	109 (16.5)	49 (15.2)	60 (17.9)	0.65
		A lot	163 (24.7)	82 (25.4)	81 (24.1)	
		Sometimes	186 (28.2)	88 (27.2)	98 (29.1)	
		Never	201 (30.5)	104 (32.2)	97 (28.9)	
During the past month, how many advertisements for cigarettes have you seen on billboards?		A lot	221 (33.5)	98 (30.3)	123 (36.6)	0.233
		A few	145 (22.0)	74 (22.9)	71 (21.1)	
		None	293 (44.5)	151 (46.8)	142 (42.3)	
During the past month, how many advertisements or promotions for cigarettes have you seen in newspapers or magazines?		A lot	193 (29.3)	101 (31.3)	92 (27.4)	<0.001
		A few	108 (16.4)	69 (21.4)	39 (11.6)	
		None	358 (54.3)	153 (47.4)	205 (61.0)	
When you go to sports events or community events, how often do you see advertisements for cigarettes?		I never attend sports events, fairs, concerts, or community events	121 (18.4)	48 (14.9)	73 (21.7)	0.108
		A lot	152 (23.1)	73 (22.6)	79 (23.5)	
		Sometimes	125 (19.0)	64 (19.8)	61 (18.2)	
		Never	261 (39.6)	138 (42.7)	123 (36.6)	
Has a cigarette representative ever offered you a free cigarette?		Yes	68 (10.3)	29 (9.0)	39 (11.6)	0.267
School activities for anti-smoking						
During this school year has a teacher or any other person ever talked in class about the dangers of smoking of smoking?		Yes	275 (41.7)	122 (37.8)	153 (46.1)	0.041
		No	258 (39.2)	142 (44.0)	116 (34.5)	
		Not sure	126 (19.1)	59 (18.3)	67 (19.9)	
During this school year, was there any discussion in any of your classes about the reasons why people your age smoke?		Yes	161 (24.4)	58 (18.0)	103 (30.7)	0.001
		No	337 (51.1)	184 (57.0)	153 (45.5)	
		Not sure	161 (24.4)	81 (25.0)	80 (23.8)	
During this school year, was there any discussion in any of your classes about the effects of smoking, like it makes your teeth yellow, it causes wrinkles, or it makes you smell bad?		Yes	201 (30.5)	73 (22.6)	128 (38.1)	<0.001
		No	333 (50.5)	182 (56.3)	151 (44.9)	
		Not sure	125 (19.0)	68 (21.1)	57 (17.0)	

Smoking preventive measures

Among those who considered quitting smoking, there was no significant difference in who could more effectively convince them to stop, with options ranging from physicians, mothers, clerics, fathers, teachers, and friends (p=0.573). However, a slightly higher percentage of respondents in the banned area strongly agreed that health institutions are responsible for convincing smokers to quit (61.6% vs. 59.2%, p=0.348), and more respondents in the banned area strongly agreed that the harmful effects of smoking should be included in the school curriculum (70.3% vs. 62.8%, p=0.047). Awareness of the existence of clinics to help smokers quit was slightly higher in the banned area (56.7% vs. 51.8%, p=0.120), and attitudes toward teachers smoking in front of students were predominantly negative in both areas, with 68.4% in the banned area and 65.5% in the non-banned area viewing it as a bad model (p=0.079), as shown in Table 7.

**Table 7:** Preventive measures and economic burden of smoking

Variables		Total (n= 659); n (%)	Banned smoking area (n=323); n (%)	Non-banned smoking area (n=336); n (%)	p-Value
Smoking preventive measures					
Who is more able to convince you to stop smoking*? (% of current smokers)	Physician	21 (16.7)	10 (21.7)	11 (13.8)	0.573
	Mother	50 (39.7)	18 (39.1)	32 (40.0)	
	Clerics	12 (9.5)	6 (13.0)	6 (7.5)	
	Father	19 (15.1)	5 (11.0)	14 (17.5)	
	Teacher	8 (6.4)	3 (6.5)	5 (6.2)	
	Friend	16 (12.7)	4 (8.7)	12 (15.0)	
Do you think that health institutions are responsible for convincing smokers to quit smoking?	Strongly agree	398 (60.4)	199 (61.6)	199 (59.2)	0.348
	Somehow agree	91 (13.8)	48 (14.9)	43 (12.8)	
	Disagree	169 (25.6)	75 (23.5)	94 (28.0)	
Do you agree that the harmful effects of smoking should be included in the school curriculum?	Strongly agree	438 (66.5)	227 (70.3)	211 (62.8)	0.047
	Somehow agree	85 (12.9)	42 (13.0)	43 (12.8)	
	Disagree	136 (20.6)	54 (16.7)	82 (24.4)	
Do you know the existence of clinics to help smokers to quit smoking?	Yes	357 (54.2)	183 (56.7)	174 (51.8)	0.120
What do you think about teachers smoking in front of students?	Bad model	441 (66.9)	221 (68.4)	220 (65.5)	0.079
	Personal freedom	69 (10.5)	30 (9.3)	39 (11.6)	
	Mimic him	33 (5.0)	22 (6.8)	11 (3.3)	
	No differences	116 (17.6)	50 (15.5)	66 (19.6)	
Economic burden of tobacco smoking					
How much you buy a cigarette package (20 cigarettes)?	Never smoke*	360 (54.6)	195 (60.4)	165 (49.1)	0.330
	Never, buy cigarettes	160 (24.3)	75 (23.2)	85 (25.3)	
	Two SR	15 (2.3)	6 (1.9)	9 (2.7)	
	3 SR	14 (2.1)	3 (0.9)	11 (3.3)	
	4 SR	3 (0.5)	1 (0.3)	2 (0.6)	
	5 SR	27 (4.1)	12 (3.6)	15 (4.5)	
	6 SR	30 (4.6)	15 (4.6)	15 (4.5)	
	>6 SR	50 (7.6)	16 (5.0)	34 (10.0)	
During the last 30 days, how much you spend money for cigarettes?	Never smoke*	360 (54.6)	195 (60.4)	165 (49.1)	0.327
	Never, buy cigarettes	160 (24.3)	75 (23.2)	85 (25.3)	
	60 SR	80 (12.1)	34 (10.5)	46 (13.7)	
	90 SR	15 (2.3)	7 (2.3)	8 (2.4)	
	120 SR	8 (1.2)	2 (0.6)	6 (1.8)	
	150 SR	8 (1.2)	2 (0.6)	6 (1.8)	
	180 SR	9 (1.4)	4 (1.2)	5 (1.5)	
	>180 SR	19 (2.9)	4 (1.2)	15 (4.4)	
What is your daily expense?	I have no daily income	27 (4.1)	5 (1.5)	22 (6.5)	<0.001
	1-2 SR	59 (9.0)	32 (9.9)	27 (8.0)	
	3-4 SR	237 (36.0)	139 (43)	98 (29.2)	
	5 SR	239 (36.3)	110 (34.1)	129 (38.4)	
	6-9 SR	31 (4.7)	16 (5.0)	15 (4.5)	
	10 SR	35 (5.3)	14 (4.3)	21 (6.3)	
	>10 SR	31 (4.7)	7 (2.2)	24 (7.1)	

Economic burden of smoking

Regarding the economic burden of tobacco smoking, there were no significant differences in the cost of cigarette packages or the amount spent on cigarettes in the last 30 days between the two areas (p=0.330 and p=0.327, respectively). However, the daily expenses of individuals showed a significant difference, with a higher percentage of respondents in the non-banned area having no daily income (6.5% vs. 1.5%, p<0.001), as shown in Table 7.

## Discussion

The findings of this cross-sectional study, indicating a significant reduction in smoking prevalence among students in areas with a smoking ban, align closely with existing literature on the impact of such public health policies. A majority of observational studies included in a systematic review suggested that school policies aimed at preventing smoking among young people can make a significant difference in reducing smoking rates among students [22]. This effect was attributed to both the reduced visibility of smoking as a normative behavior and the limited access to environments where smoking is permissible.

Additionally, the observed reduction in the frequency of smoking behaviors among students in the banned area is consistent with the literature. According to El Amin's study, anti-smoking policies in schools can reduce the prevalence of smoking by promoting prevention, restriction, cessation, and preventing students from starting smoking in the first place [23]. It was reported that students' compliance with smoking bans in schools is generally high, with a study reporting that 72.7% of students were compliant with the ban, and for some, it led to a decrease in their smoking habits [24]. Not only do these bans reduce the prevalence of smoking among students, but they also play a crucial role in altering smoking behavior, leading to less frequent usage.

The influence of family and peers on smoking habits is also reduced in the banned area, indicating a broader impact of the smoking ban on the social environment of these students. A meta-analysis of 61 studies showed that parental and peer smoking behaviors are strong predictors of adolescent smoking initiation. However, the study also noted that comprehensive smoking bans can mitigate these influences by changing social norms around smoking [25]. The reduction in smoking visibility due to such bans may decrease the modeling of smoking behavior by family members and peers, thereby lessening their influence on adolescents.

Furthermore, the increase in discussions about the harmful effects of smoking in families from the banned area is a noteworthy observation. It was suggested that public health interventions like smoking bans often extend beyond their immediate context, prompting broader societal discussions about health behaviors [26]. In the case of smoking bans, these discussions likely reinforce the dangers of smoking, particularly in families where adolescents are present, further discouraging smoking initiation and continuation among young individuals.

The differences in accessibility and attitudes toward smoking observed in the study are crucial in understanding the complex dynamics of smoking behavior among students. Access to tobacco products plays a crucial role in the prevalence of smoking, especially in areas where regulations are not stringent [27]. Another study identified access to tobacco products as one of the leading contributors to smoking initiation among teenagers [28]. An Egyptian study showed that in areas where access is more restricted, smoking rates tend to be lower [29].

Regarding attitudinal differences, the study's observation that students in the banned area are more optimistic about not smoking in the future, despite holding more positive social perceptions of smoking, is particularly intriguing. This paradox might be explained by the "forbidden fruit" effect [30]. Additionally, the improved recognition of health perceptions regarding the harmful effects of smoking in the banned area resonates with findings from a study by Zhu et al., which found that increased exposure to anti-smoking messages and environments, such as those created by smoking bans, enhances young people's understanding of the health risks associated with smoking [31].

Passive smoking exposure is another critical aspect covered in the study. Students in the non-ban area report moderate exposure both at home and in other places, indicating a more pervasive smoking culture in their immediate environments [32]. This increased exposure to passive smoking in non-banned areas has significant implications for public health, including the risk of respiratory problems and cardiovascular issues [33].

Regarding cessation efforts among current smokers, there are indications of a higher desire to quit in the banned area, although the differences are not statistically significant. Media and school activities related to anti-smoking messages are more prevalent in the banned area, which might be influencing these attitudes and behaviors. Finally, the economic burden of smoking does not significantly differ between the two areas. However, the study notes differences in daily expenses and income levels, providing insight into the economic context of smoking behaviors.

Future directions

The findings of this study offer valuable insights into the impact of smoking bans in schools and their influence on adolescent smoking behavior. Future research should aim to explore longitudinal data to better understand the long-term effects of these bans. Additionally, investigating the mechanisms through which smoking bans lead to changes in smoking behaviors could provide deeper insights. For instance, studies could examine the role of changing social norms, increased health awareness, and reduced accessibility in influencing adolescent smoking habits. It is also important to explore the effectiveness of such bans in diverse cultural and socioeconomic settings to understand their broader applicability.

Limitations

This study, while comprehensive, has several limitations. The cross-sectional design limits the ability to infer causality between smoking bans and observed behavioral changes. Furthermore, the reliance on self-reported data may introduce biases, as students might underreport or overreport their smoking habits due to social desirability or recall issues. Additionally, the study was conducted in only two schools, which may limit the generalizability of the findings to other settings. Finally, the study did not account for other concurrent anti-smoking measures, such as public health campaigns or parental interventions, which might have influenced the results.

## Conclusions

This study highlights the significant benefits of implementing smoking bans in schools. Our findings demonstrate a notable reduction in smoking prevalence among students in areas with such bans. These policies not only diminish the visibility and accessibility of smoking but also alter students' attitudes toward tobacco use. The influence of family and peers, major determinants of adolescent smoking behavior, is mitigated in these environments, indicating a broader impact of smoking bans. Furthermore, discussions about the harmful effects of smoking have increased in families in ban areas, suggesting a positive societal shift. The study's insights emphasize the need for comprehensive smoking bans in schools as a crucial component of public health strategies to combat smoking among youth. However, there remains an unmet need for more targeted interventions that address the complexities of smoking behavior, particularly in environments without such bans. This includes addressing attitudinal differences, improving accessibility restrictions, and enhancing educational efforts on the harms of tobacco use. Our research underscores the importance of continued efforts in these areas to ensure the well-being of future generations.
